# Risk factors, impact on outcomes, and molecular epidemiology of infections caused by carbapenem-resistant *Enterobacterales* in intensive care patients: a multicenter matched case–control study in Brazil

**DOI:** 10.62675/2965-2774.20250237

**Published:** 2025-04-02

**Authors:** Pedro Fernandez Del Peloso, Pedro Kurtz, Bianca Brandão de Paula Antunes, Leonardo dos Santos Lourenço Bastos, Silvio Hamacher, Fernando Augusto Bozza

**Affiliations:** 1 Instituto D’Or de Pesquisa e Ensino Rio de Janeiro RJ Brazil Instituto D’Or de Pesquisa e Ensino - Rio de Janeiro (RJ), Brazil.; 2 Pontifícia Universidade Católica do Rio de Janeiro Department of Industrial Engineering Rio de Janeiro RJ Brazil Department of Industrial Engineering, Pontifícia Universidade Católica do Rio de Janeiro - Rio de Janeiro (RJ), Brazil.; 3 Instituto Estadual do Cérebro Paulo Niemyer Neurological Intensive Care Unit Rio de Janeiro RJ Brazil Neurological Intensive Care Unit, Instituto Estadual do Cérebro Paulo Niemyer - Rio de Janeiro (RJ), Brazil.

**Keywords:** Enterobacterales, Carbapenem, Carbapenem-resistant Enterobacteriaceae, Klebsiella pneumoniae, Infections, Sepsis, Hospital mortality, Molecular epidemiology, Genotype, Risk factors, Intensive care units

## Abstract

**Objective:**

To evaluate risk factors, molecular profiles, and hospital mortality of carbapenem-resistant *Enterobacterales* (CRE) infections in intensive care unit patients.

**Methods:**

In this retrospective, multicenter cohort study, intensive care unit admissions from 52 intensive care units between January 2019 and December 2020 were analyzed in a nested case-control design. Patients with carbapenem-resistant *Enterobacterales* infections were propensity score-matched 1:1 to those with carbapenem-susceptible *Enterobacterales* infections. Hierarchical conditional logistic regression identified risk factors for carbapenem-resistant *Enterobacterales* , and multivariable logistic regression assessed the association of carbapenem-resistant *Enterobacterales* with 60-day in-hospital mortality. Molecular genotyping was also conducted.

**Results:**

Matching resulted in 250 carbapenem-resistant *Enterobacterales* patients and 250 carbapenem-susceptible *Enterobacterales* patients. Sepsis was more common in the carbapenem-resistant *Enterobacterales* group (58% *versus* 35%; p < 0.001). Risk factors for carbapenem-resistant *Enterobacterales* included major premorbid assistance requirements (OR 1.72, 95%CI 0.99 - 3.01; p = 0.06) and intensive care unit readmission (OR 1.87, 95%CI 1.00 - 3.49; p = 0.05), although with weak associations. Acute COVID-19 (OR 3.55, 95%CI 1.96 - 6.45; p < 0.001) also increased the odds of resistance. Carbapenem-resistant *Enterobacterales* infection was associated with twice the likelihood of 60-day mortality after adjusting for covariates (OR 1.95, 95%CI 1.26 - 3.02; p < 0.001). The predominant bacteria and carbapenemase resistance genes included *Klebsiella pneumoniae* (79%), *Klebsiella pneumoniae* carbapenemase (73%), New Delhi metallo-beta-lactamase (13%), and xacillinase-48 (9%).

**Conclusion:**

Carbapenem-resistant *Enterobacterales* -related infections in intensive care unit patients were associated with major premorbid dependence, intensive care unit readmission, and acute COVID-19. In addition, carbapenem-resistant *Enterobacterales* infections were independently associated with poorer hospital outcomes. This study also characterized the resistance profile of Enterobacterales in Brazilian intensive care units, which are dominated by *K. pneumoniae* with high rates of carbapenemase and increased rates of New Delhi metallo-beta-lactamase, in comparison with previous reports.

## INTRODUCTION

Increased rates of carbapenem-resistant *Enterobacterales* (CRE) infection are concerning in patients with community- and hospital-acquired severe infections.^( 1 )^ Risk factors for CRE infections are poorly understood, and treatment alternatives are limited, which hampers effective empirical antimicrobial therapy options.^( 2 , 3 )^ Carbapenem-resistant *Enterobacterales* are considered a critical priority for research and drug development by the World Health Organization (WHO),^( 4 )^ especially in low- and middle-income countries where mortality due to sepsis remains elevated.^( 5 )^ In Brazil in particular, nationwide research efforts are ongoing to better understand the scope of the problem and design potential mitigating strategies and clinical trials.^( 3 )^ The detection of resistance to carbapenems, as well as the correct identification of these resistance mechanisms, are of vital importance in the selection of adequate antimicrobial therapy, which directly affects the clinical cure rate and infection prognosis. Our study aimed to define the risk factors, molecular and clinical profiles, and impact on 60-day hospital mortality due to CRE infection in patients admitted to intensive care in a nested case–control multicenter study.

## METHODS

### Study design, participants, sites, and study period

We performed a retrospective analysis of intensive care unit (ICU) admissions from an integrated hospital network. The data included admissions from January 1, 2019, to December 31, 2020. We included a convenience sample of all consecutive adult patients admitted to 52 ICUs distributed across 8 hospitals in Rio de Janeiro, Brazil. The Local Ethics Committee and the Brazilian National Ethics Committee (CAAE: 17079119.7.0000.5249) approved the study without the need for informed consent. The participating sites included consecutive patients diagnosed with target infections (urinary tract infections, intraabdominal infections, pneumonia, and bloodstream infections from other sources) caused by CRE. For this analysis, a nested matched case–control study design was used. Patients with CRE infections were eligible as cases, and the control group included patients with infections caused by carbapenem-susceptible *Enterobacterales* (CSE) selected from the larger cohort. Patients in the control group were matched to those in the CRE group for the following variables: hospital admission, source of ICU admission (emergency, operating theater, transfers), cultured sample (blood, respiratory, urine, others), length of hospital stay prior to culture, and Simplified Acute Physiology Score 3 (SAPS 3) ( Table 1 ).

**Table 1:** Characteristics and organ support of matched patients with carbapenem-sensitive and carbapenem-resistant *Enterobacterales*

Characteristic			Overall N = 500	CRE n = 250	CSE n = 250	p value
Matching variables						
	Source of ICU admission					0.8
		Emergency room	246 (49)	127 (51)	119 (48)	
		Operating room or catheterization laboratory Cathlab	51 (10)	24 (9.6)	27 (11)	
		Transfer from ward or other hospital	203 (41)	99 (40)	104 (42)	
	Type of ICU admission					> 0.9
		Emergency surgery	21 (4.2)	10 (4.0)	11 (4.4)	
		Medical	444 (89)	223 (89)	221 (88)	
		Scheduled surgery	35 (7.0)	17 (6.8)	18 (7.2)	
	Sample					0.6
		Blood	90 (18)	41 (16)	49 (20)	
		Others	43 (8.6)	18 (7.2)	25 (10)	
		Respiratory	214 (43)	113 (45)	101 (40)	
		Urine	142 (28)	72 (29)	70 (28)	
		Length of stay before culture	9 (3 - 17)	10 (4 - 16)	9 (2 - 18)	0.6
		SAPS 3	57 (48 - 69)	58 (49 - 68)	56 (47 - 69)	0.2
Nonmatching variables						
	Age		73 (61 - 83)	72 (61 - 82)	74 (62 - 85)	0.2
	Female		250 (50)	115 (46)	135 (54)	0.07
	Comorbidities					
		Malignancy	120 (24)	61 (24)	59 (24)	0.8
		Immunosuppression	130 (26)	59 (24)	71 (28)	0.2
		Hypertension	347 (69)	172 (69)	175 (70)	0.8
		Diabetes	196 (39)	100 (40)	96 (38)	0.7
	Frailty					0.9
		Nonfrail (MFI = 0)	62 (12)	31 (12)	31 (12)	
		Prefrail (MFI = 1 - 2)	243 (49)	124 (50)	119 (48)	
		Frail (MFI ≥ 3)	195 (39)	95 (38)	100 (40)	
	Performance status					0.4
		Ambulant/independent	278 (56)	132 (53)	146 (58)	
		Minor assistance	113 (23)	58 (23)	55 (22)	
		Major assistance/bedridden	109 (22)	60 (24)	49 (20)	
	Primary admission diagnosis					< 0.001
		Infection/sepsis	232 (46)	144 (58)	88 (35)	
		Cardiovascular	63 (13)	22 (8.8)	41 (16)	
		Other diagnoses	205 (41)	84 (33)	121 (49)	
		Readmission in the hospital	63 (13)	38 (15)	25 (10)	0.08
	At admission					
		Glasgow coma scale at admission	15 (10 - 15)	14 (10 - 15)	15 (10 - 15)	0.06
		MV at ICU admission	186 (37)	102 (41)	84 (34)	0.10
		Vasopressor at ICU admission	177 (35)	96 (38)	81 (32)	0.2
		Hemodalysis at ICU admission	54 (11)	32 (13)	22 (8.8)	0.15
		SOFA score	3 (1 - 8)	3 (1 - 8)	2 (0 - 8)	0.06
	During ICU stay					
		MV	321 (64)	180 (72)	141 (56)	< 0.001
		Vasopressors	264 (53)	148 (59)	116 (46)	0.004
		Hemodialysis	144 (29)	83 (33)	61 (24)	0.03
		Acute COVID-19	86 (17)	63 (25)	23 (9.2)	< 0.001

CRE - carbapenem-resistant *Enterobacterales* ; CSE - carbapenem-susceptible *Enterobacterales* ; ICU - intensive care unit; SOFA - Sequential Organ Failure Assessment; MFI - modified frailty index. Data are presented as medians and interquartile ranges (25 - 75%) or n (%).

### Variables, data collection and quality control

We collected anonymized data from an Epimed Monitor^®^ electronic system used for benchmarking purposes (Rio de Janeiro, Brazil).^( 6 )^ It contains information regarding the patients’ characteristics, comorbidities, frailty,^( 7 )^ premorbid performance status, primary diagnosis at ICU admission, severity scores, such as the SAPS 3,^( 8 )^ organ support in the ICU (mechanical ventilation [MV], vasopressor use, and renal replacement therapy), and hospital outcomes. We included only complete cases with outcomes. We also analyzed the database retrieved from the hospitals’ laboratory systems, which contain the patient's identification number and microbiology results.

### Microbiological definitions and studies

*Enterobacterales* isolates with resistance to carbapenems were identified at the genus and species levels via the VITEK^®^ MS MALDI-TOF mass spectrometry methodology (bioMérieux - France). The initial determination of resistance to carbapenems was performed by automated equipment Vitek^®^ 2 XL (bioMérieux – France) together with the AST-N239 susceptibility card, where ertapenem, imipenem and meropenem were tested. The results with carbapenem resistance phenotypes obtained by the Vitek^®^ 2 XL equipment were confirmed via the Kirby–Bauer disk-diffusion reference methodology (Brazilian Committee on Antimicrobial Susceptibility - BrCAST), and the minimum inhibitory concentration was determined via a diffusion gradient strip (Etest^®^).^( 9 )^ For the results of resistance phenotypes confirmed by reference methodology for any of the carbapenems, detection of carbapenemase production was performed by a fast colorimetric method using the RAPIDEC^®^ Carba NP test (bioMérieux – France).^( 10 )^ Isolates that were carbapenemase positive by the colorimetric method had their carbapenemase genes characterized by polymerase chain reaction (PCR) with PCR amplification of resistance genes, followed by reverse hybridization (dot blot) using specific immobilized DNA probes in a nylon membrane (CHIP (XGEN MULTI SEPSE CHIP^®^ - Mobius Life Science)), which detected the following broad-spectrum carbapenemase and beta-lactamase genes: KPC, NDM, SME, NMC/IMI, BLASHV, BLACTX-M, GES, VIM, GIM, SPM, SIM, IMP, OXA-48, OXA-23, OXA-24, OXA-51 and OXA-58.^( 11 )^

### Statistical analysis

Data are presented as medians and interquartile ranges (IQRs 25 - 75%) for continuous variables and as absolute numbers and percentages for categorical variables. Univariate associations were tested by using the chi-square test or Fisher's exact test for categorical variables and the Mann–Whitney U test for continuous variables.

**Figure 1:**
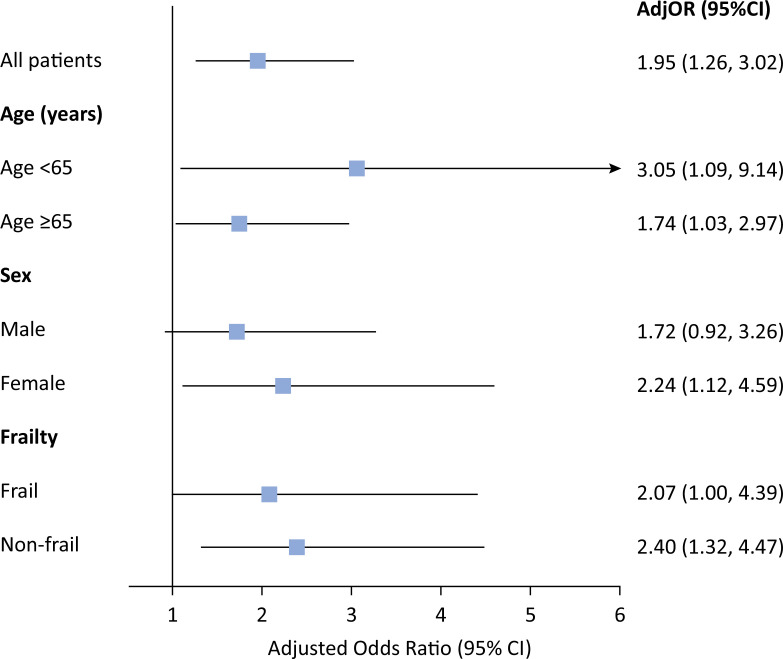
Multivariable logistic regression analysis of factors associated with 60-day mortality in matched patients with carbapenem-susceptible *Enterobacterales* and carbapenem-resistant *Enterobacterales* infections.

For the analysis of risk factors among CRE and CSE patients, hierarchical conditional logistic regression was performed for matched data, which considers the matched strata, controlling for potential confounders in the matching process ( Figure 1S - Supplementary Material ). Matching was performed using propensity scores estimated using a logistic regression model with CRE status as the response variable and the aforementioned matching variables as predictors.^( 12 )^ The matching procedure utilized the nearest neighbor with a caliper of 0.25 in a 1:1 fashion. Selection of variables included in the multivariable models was performed a priori on the basis of clinical relevance.

Associations between 60-day hospital mortality and CRE infection were evaluated with multivariable logistic regression with carbapenem resistance as the main exposure variable and mortality as the binary outcome variable with potential confounders adjusted for. We present the results of the full models, with covariates included on the basis of clinical relevance and chosen a priori. We also present sensitivity analyses in subgroups of age, sex, and frailty status, which were chosen a priori by the research team. The analyses were performed using R (R Core Team, 2024, Version 4.3.2).^( 13 )^

## RESULTS

### Risk factors for carbapenem-resistant *Enterobacterales* infection

Overall, 500 patients were matched, and comparisons were made between two groups: infections due to CRE and those due to CSE. The full cohort included 2,487 patients, 250 of whom fulfilled the inclusion criteria for infection due to CRE. Age, sex, and premorbid status did not differ between the groups, whereas sepsis was a more frequent admission diagnosis in the CRE group (58% *versus* 35%; p < 0.001) ( Table 1 ). The rates of organ dysfunction and support, measured by the SOFA score and the use of MV, vasopressors, and hemodialysis, were similar between the groups at admission. During the ICU stay, however, organ support requirements were more common in the CRE group than in the CSE group (ventilation 72% *versus* 56%; p < 0.001; vasopressors 59% *versus* 46%; p = 0.004; hemodialysis 33% *versus* 24%; p = 0.030, respectively). Moreover, patients who developed infection with CRE were more likely to have concomitant acute COVID-19 than were those with CSE (25% *versus* 9%; p < 0.001). In the multivariable hierarchical conditional logistic regression analysis ( Table 2 ), the risk factors that weakly associated with CRE-related infection in the ICU were premorbid functional status (major assistance *versus* no assistance, odds ratio [OR] OR 1.72, 95%CI 0.99 - 3.01; p = 0.06), readmission to the ICU (OR 1.87, 95%CI 1.00 - 3.49; p = 0.05), and acute COVID-19 (OR 3.55, 95%CI 1.96 - 6.45; p < 0.001).

**Table 2:** Multivariable hierarchical conditional logistic regression analysis of risk factors for carbapenem-resistant *Enterobacterales* infection

Parameter		Odds ratio	95%CI	p value
Age		1.00	0.98 - 1.01	0.59
Female sex		1.18	0.79 - 1.76	0.43
Frailty				
	Nonfrail (MFI = 0)	1	-	-
	Prefrail (MFI = 1 - 2)	1.04	0.49 - 2.22	0.91
	Frail (MFI ≥ 3)	0.78	0.31 - 1.95	0.6
Performance status				
	Ambulant/independent	1	-	-
	Minor assistance	1.64	0.95 - 2.82	0.08
	Major assistance/bedridden	1.72	0.99 - 3.01	0.06
At ICU admission				
	MV	1.36	0.73 - 2.55	0.3
	Vasopressors	1.04	0.57 - 1.90	0.9
	Hemodialysis	1.23	0.63 - 2.42	0.55
Readmission to the ICU		1.87	1.00 - 3.49	0.05
Comorbidities				
	Asthma/COPD	1.14	0.59 - 2.17	0.7
	Immunosuppression	0.80	0.51 - 1.27	0.34
	Acute COVID-19	3.55	1.96 - 6.45	< 0.001

MFI - modified frailty index; ICU - intensive care unit; MV - mechanical ventilation; COPD - chronic obstructive pulmonary disease.

### Association between carbapenem-resistant *Enterobacterales* infection and hospital mortality

The primary outcome of hospital mortality at 60 days was higher in the CRE group (CRE 46% *versus* CSE 29%; p < 0.001). Secondary outcomes such as ICU mortality and ICU LOS were also increased in the CRE group (CRE 42% *versus* CSE 27%; p < 0.001 and CRE 17 days [8 - 29] *versus* CSE 13 days [5 - 27]; p = 0.037, respectively) ( Table 3 ). In the multivariable multilevel analysis ( Figure 1 ), after adjusting for relevant covariates, patients with CRE-related infections were twice as likely to die at 60 days than were those with CSE infection (OR 1.95, 95%CI 1.26 - 3.02; p < 0.001). Sensitivity analyses revealed similar results across ages younger and older than 65 years, while the association was more prominent in nonfrail (OR 2.4, 95%CI 1.32 - 4.47) and female patients (OR 2.24, 95%CI 1.12 - 4.59).

**Table 3:** Hospital complications and outcomes in matched patients with carbapenem-sensitive and carbapenem-resistant *Enterobacterales*

Characteristic		N	Overall n = 500	CRE n = 250	CSE n = 250	p value
Invasive MV		500	234 (47)	132 (53)	102 (41)	0.007
Duration of hemodialysis		144	13 (8 - 19)	14 (8 - 21)	13 (8 - 16)	0.3
Duration of intravenous short-term catheter		321	19 (10 - 28)	19 (11 - 28)	19 (10 - 28)	0.8
Duration of invasive MV		234	16 (6 - 26)	15 (6 - 24)	17 (7 - 27)	0.3
Length of stay						
	ICU	500	15 (7 - 28)	17 (8 - 29)	13 (5 - 27)	0.04
	Hospital	500	31 (18 - 56)	30 (20 - 54)	31 (16 - 58)	0.5
Mortality						
	ICU	500	173 (35)	106 (42)	67 (27)	< 0.001
	Hospital	500	234 (47)	138 (55)	96 (38)	< 0.001
	30-day	500	119 (24)	77 (31)	42 (17)	< 0.001
	60-day	500	188 (38)	116 (46)	72 (29)	< 0.001

CRE - carbapenem-resistant *Enterobacterales* ; CSE - carbapenem-susceptible *Enterobacterales* ; MV - mechanical ventilation; ICU - intensive care unit. Data are presented as n (%) or medians and interquartile ranges (25 - 75%).

The adjusted odds ratio for "All patients" reflects the positive independent association of carbapenem-resistant *Enterobacterales* with increased mortality in the main model with the fully matched cohort. Sensitivity analyses of elderly and nonelderly individuals, men and women, and the presence or absence of frailty are also presented. The models were adjusted for admission source, age, sex, frailty, premorbid functional status, acute organ support requirements (mechanical ventilation, vasopressors, hemodialysis), readmission, Glasgow coma score, comorbidities, and COVID-19 status.

### Molecular epidemiology

**Figure 2:**
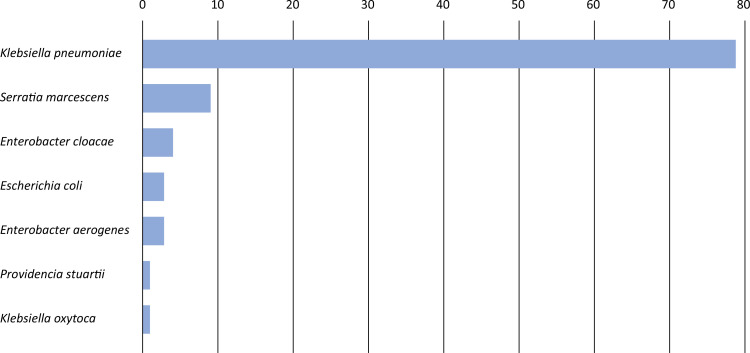
Carbapenem-resistant *Enterobacterales* (number of samples studied).

**Figure 3:**
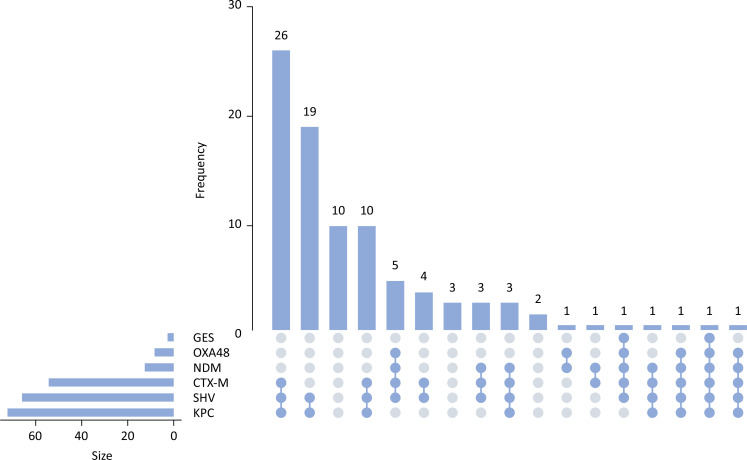
Molecular mechanisms of resistance. Attribute plot illustrating isolated and combined molecular profiles of resistance mechanisms among the 100 samples examined.

We studied 100 clinical samples: blood (52%) and samples from the respiratory tract (7%), among others (41%). The bacterial prevalence rates were as follows: *Klebsiella pneumoniae,* 79%; *Serratia marcescens,* 9%; *Enterobacter cloacae,* 4%; *Enterobacter aerogenes,* 3%; *Escherichia coli,* 3%; *Klebsiella oxytoca,* 1%; and *Providencia stuartii,* 1% ( Figure 2 ). The following (and broad-spectrum beta-lactamase) genes were detected: *Klebsiella pneumoniae* carbapenemase (KPC, 73%), New Delhi metallo-beta-lactamase (NDM, 13%), oxacillinase-48 OXA-48 (9%), Guiana extended-spectrum beta-lactamase (GES) (2%), SHV (67%), and cefotaximase-Munich beta-lactamase (CTX-M) (55%) ( Figure 3 ). The following genes were not detected: SME, NMC/IMI, VIM, GIM, SPM, SIM, IMP, OXA-23, OXA-24, OXA-51, and OXA-58. Eight isolates were carbapenem resistant with positive colorimetric detection for carbapenemase without carbapenem genes, and six isolates were carbapenem resistant without carbapenemase genes and without carbapenemase production according to colorimetric tests. In these six isolates, only broad-spectrum beta lactamase genes (CTX-M and/or SHV) were detected ( Tables 1S and 2S - Supplementary Material ).

The frequency axis shows the number of combined profiles of genes. The size axis shows the number of individual genes identified. GES - Guiana extended-spectrum beta-lactamase. OXA-48 - Oxacillinase-48; NDM - New Delhi metallo-beta-lactamase; CTX-M - cefotaximase-Munich beta-lactamase; KPC - *Klebsiella pneumoniae* carbapenemase.

## DISCUSSION

Our study identified risk factors for the development of CRE-related infection, namely, premorbid major dependence, ICU readmission, and exposure to COVID-19. Moreover, we found that infection due to CRE was independently associated with hospital outcomes, even after adjusting for patient profiles and disease severity. Finally, we described the microbiological and molecular profiles of *Enterobacterales* that are resistant to carbapenems in the ICU.

In contrast to prior large studies that reported colonization and antibiotic exposure as the main risk factors for CRE, our data did not include those variables. Our findings concerning risk factors, including the requirement for major assistance for activities of daily living and ICU readmission, are complimentary and may be interpreted as potential surrogates for prior antibiotic exposure. Nevertheless, these findings can guide institutions and physicians in identifying patients at risk of developing CRE-related infections. COVID-19 was also an independent risk factor. Various studies have shown that patients admitted for COVID-19 during the pandemic received more unnecessary antibiotics, which has also been linked to an increase in resistance patterns, especially in the ICU.^( 14 )^ Antimicrobial stewardship programs, with a special focus on high-risk critically ill patients, are essential to mitigate the threat of CRE in the ICU.

We also showed that the resistance pattern of infections (CRE *versus* CSE) was associated with varying hospital outcomes of ICU patients. This may be related to various factors, including inappropriate empiric antimicrobial choices, delays in the correct identification of microbial etiology, or more virulent strains of *Enterobacterales* . Furthermore, carbapenem resistance has increasingly been identified as a marker of multidrug resistance, further complicating the treatment and clinical management of these patients.^( 15 )^ Compared with infections caused by CSE organisms, infections caused by CRE organisms are often associated with higher mortality rates, longer hospital stays, and increased health care costs.^( 16 , 17 )^

This difference in outcomes can be partially explained by the limited ability of alternative antimicrobials to penetrate the site of infection, the use of which is necessary when carbapenems are not an effective antimicrobial option.^( 16 , 17 )^ Furthermore, CRE infections tend to occur in patients with a greater burden of comorbidities and are already in a more fragile state of health, thus increasing the risk of adverse outcomes.^( 18 )^ Choosing the appropriate antimicrobial regimen is crucial and should be based not only on the pathogen's susceptibility but also on the individual characteristics of the patient, such as renal function, potential for drug interactions, and allergy history.^( 19 )^

The emergence of carbapenem resistance also underscores the need for more robust infection prevention and control strategies in hospital settings, especially in intensive care units, where the transmission risk of multidrug-resistant organisms is particularly high.^( 18 )^ Continuous monitoring of resistance patterns and strict adherence to infection control practices are essential to mitigate the spread of these challenging pathogens.^( 20 )^

The most prevalent carbapenemase-producing gene was KPC, with the NDM gene being the second most prevalent, which is concerning in comparison to previous reports that reported much lower rates in Brazilian institutions.^( 1 , 21 )^ The coexistence of different genes in the same isolate, both carbapenemase and broad-spectrum beta lactamase genes, demonstrates plasmid dissemination among isolates, further complicating empirical antimicrobial therapy at these institutions. Carbapenemase genes such as bla_KPC, bla_NDM-like, bla_VIM-like, blaOXA-48-like and bla_IMP-like play significant roles in carbapenem resistance. In a study analyzing 1,700 *Klebsiella pneumoniae* isolates, 682 isolates carried one or more carbapenemase genes. Among these, bla_KPC-like was found in 311 isolates, blaOXA-48-like 248, NDM-like in 79, bla_IMP-like in 3 isolates and blaVIM-like in 56 isolates.^( 22 )^

In addition to carbapenemase genes, resistance mechanisms in *Enterobacterales* often involve the presence of broad-spectrum beta-lactamases, such as extended-spectrum beta-lactamases (ESBLs) and AmpC beta-lactamases. These enzymes hydrolyze most beta-lactam antibiotics, including penicillins, cephalosporins, and, in the case of ESBLs, carbapenems.^( 22 )^

Clonal dissemination and horizontal transfer of resistance genetic elements are key factors in the spread of resistance to carbapenems. The ability of *Enterobacterales* to accumulate multiple resistance genes, either through chromosomal mutations or through the acquisition of plasmids, further complicates the control of these infections. For example, *K. pneumoniae* can acquire resistance to carbapenems both through the production of carbapenemase and through changes in the pores of the outer membrane, reducing its permeability to the antibiotic.^( 22 )^

Molecular epidemiological surveillance and understanding of these resistance mechanisms are essential for developing effective strategies for controlling and treating carbapenem-resistant *Enterobacterales* infections. This includes the identification and monitoring of resistant strains, as well as the implementation of rational antimicrobial use policies and antimicrobial stewardship programs to prevent the spread of multidrug-resistant organisms.^( 23 )^

Our study has several limitations. First, we did not evaluate important factors, such as prior colonization and antibiotic exposure, as potential risk factors for CRE infection. Nonetheless, we found additional information on premorbid conditions related to acute admission that may be used by institutions and physicians to identify high-risk patients. Second, the clinical criteria for infection and antimicrobial therapy were not available in the database and not analyzed, which would have been important to avoid the inclusion of cases of colonization and to adjust the association between CRE-related infection and outcomes. Third, potential selection bias may have been present since the isolates were not genotypically discriminated, making it impossible to determine the sequence type or assess the presence of the same clone. Therefore, we were unable to characterize prevalent sequence types and compare them with other similar studies elsewhere in the world and their genotypic characteristics. There is also the possibility of not detecting other carbapenemase genes due to the limitations of the genes investigated in the present study, which could explain some unexpected results, especially in *Serratia marcescens* isolates in which carbapenemase was detected via colorimetric tests, which are resistant to meropenem but lack the presence of a carbapenemase gene. Fourth, gradient diffusion strips (Etest^®^) of antibiotics were used to determine the minimum inhibitory concentration and confirm carbapenem resistance. The standard method for this determination is broth microdilution, but the methodology used has excellent correlation with the standard methodology demonstrated in the literature.^( 9 )^ Finally, we analyzed only the first infection caused by *Enterobacterales* in our study. We cannot exclude those patients who had other subsequent infections with CSE or CRE during their hospitalization or after hospital discharge, which could affect our results.

## CONCLUSION

In our matched sample, patients with premorbid major dependence, those who underwent intensive care unit readmission, and those with acute COVID-19 were at increased risk for carbapenem-resistant *Enterobacterales* -related infections. In addition, infections caused by resistant *Enterobacterales* were independently associated with a two-fold increase in odds of death in the hospital compared with carbapenem-susceptible *Enterobacterales* infections, even after matching and adjusting for relevant confounders. Moreover, resistant *Enterobacterales* presented distinct strains and molecular profiles, with frequent detection of *Klebsiella pneumoniae* carbapenemase and New Delhi metallo-beta-lactamase genes, highlighting the importance of rapid genotyping for appropriate antimicrobial therapy.
