# Supplementary material for: Risk factors, impact on outcomes, and molecular epidemiology of infections caused by carbapenem-resistant Enterobacterales in intensive care patients: a multicenter matched case–control study in Brazil
Source: Crit Care Sci. 2025 Apr 2;37:e20250237. doi: 10.62675/2965-2774.20250237 (PMC12040421; doi:10.62675/2965-2774.20250237)
Supplement: Supplementary file 1 [file 2965-2774-ccsci-37-e20250237-Mat-suppl.pdf]

Critical Care Science
https://doi.org/10.62675/2965-2774.20250237

Risk factors, impact on outcomes, and molecular epidemiology of infections caused by carbapenem-resistant *Enterobacterales* in intensive care patients: a multicenter matched case–control study in Brazil

Pedro Fernandez Del Peloso[1], Pedro Kurtz[1], Bianca Brandão de Paula Antunes[2], Leonardo dos Santos Lourenço Bastos[2], Silvio Hamacher[2], Fernando Augusto Bozza[3]

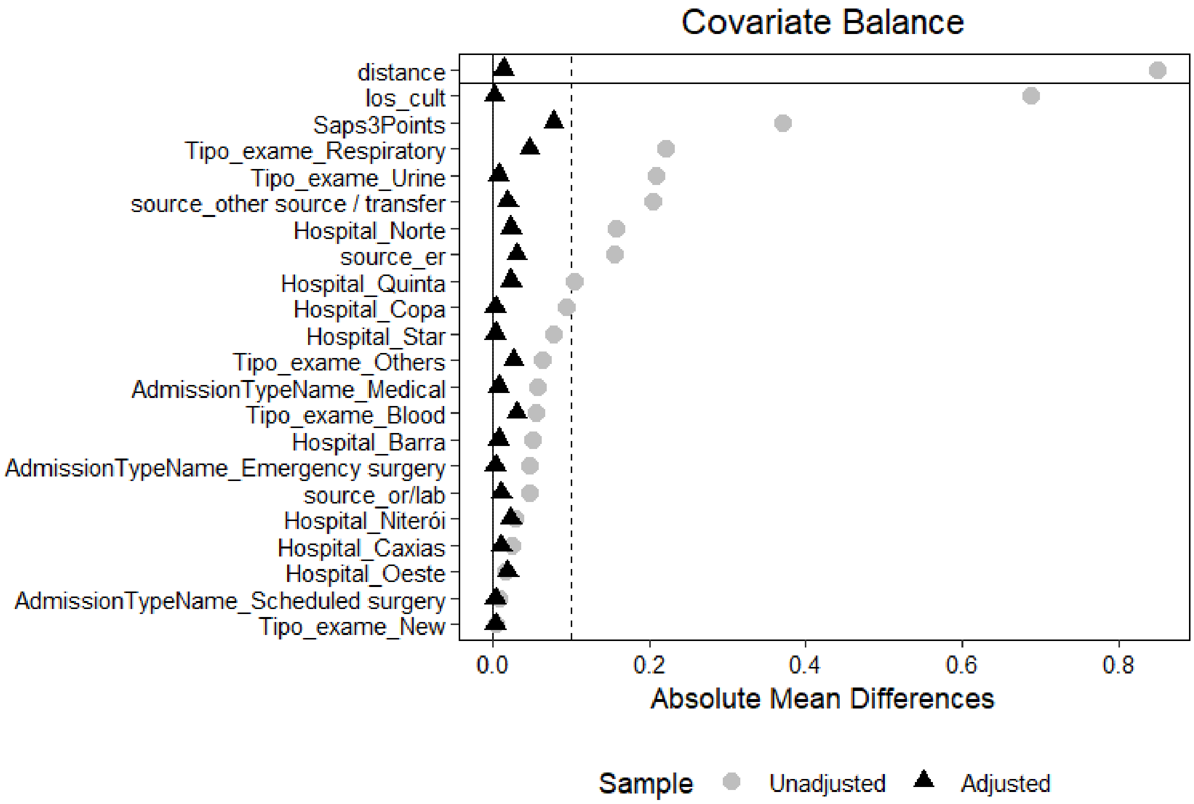

Figure 1S - Love plot with measures of covariate balance distribution before and after adjustment using standardized mean differences. Values below 0.1 are considered adequate.

This is an open access article under the CC BY license (https://creativecommons.org/licenses/by/4.0/).

Table 1S - Main types of carbapenemases

Carbapenemase type	Ambler class	Enzyme examples	Mechanism of action	Inhibition	Detection challenges
KPC	A	KPC	Hydrolyzes a wide range of β-lactams, including carbapenems	Resistant to conventional β-lactamase inhibitors	None significant
MBL	B	NDM, VIM, IMP	Uses zinc ions to hydrolyze β-lactams	Inhibited by zinc-chelating agents	Requires specific chelating agents for inhibition
OXA-48	D	OXA-48	Hydrolyzes carbapenems with lower efficiency	Difficult to detect due to low activity against some carbapenems	Low detection sensitivity

KPC - *Klebsiella pneumoniae* carbapenemase; NDM - New Delhi metallo-beta-lactamase; OXA-48 - Oxacillinase-48; VIM - Verona integron-encoded metallo-beta-lactamase; IMP - Imipenemase metallo-beta-lactamase.

Table 2S - Carbapenemases found per bacteria species

Bacterial identification	KPC	VIM	IMP3	OXA-48	NDM	GES
Enterobacter aerogenes	3	0	0	0	0	0
Enterobacter cloacae	4	0	0	1	1	0
Escherichia coli	2	0	0	0	2	0
Klebsiella oxytoca	1	0	0	0	0	0
Klebsiella pneumoniae	75	0	0	7	7	2
Providencia stuartii	0	0	0	0	1	0
Serratia marcescens	5	0	0	0	1	0

KPC - *Klebsiella pneumoniae* carbapenemase; VIM - Verona integron-encoded metallo-beta-lactamase; IMP - Imipenemase metallo-beta-lactamase; OXA-48 - Oxacillinase-48; NDM - New Delhi metallo-beta-lactamase; GES - Guiana extended-spectrum beta-lactamase.
